# Rotationally Resolved Infrared Spectroscopy of Supersonic Jet-Cooled Isoprene

**DOI:** 10.1021/acs.jpca.3c02272

**Published:** 2023-05-26

**Authors:** Jacob T. Stewart, Lauren Hino, Carter Pavlonnis, Katarina R. Reyna, Binh L. N. Vo

**Affiliations:** Department of Chemistry, Connecticut College, New London, Connecticut 06320, United States

## Abstract

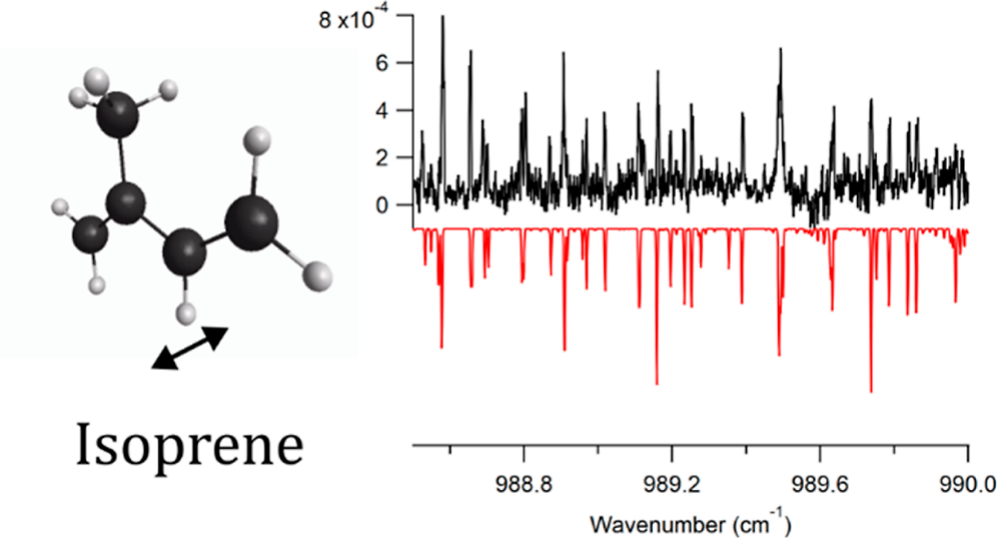

The high-resolution infrared spectrum of isoprene has been observed under supersonic jet-cooled conditions in the region of the ν_26_ vibrational band near 992 cm^–1^. The spectrum was assigned and fit using a standard asymmetric top Hamiltonian, and an acceptable fit was obtained for transitions to excited state energy levels with *J* ≤ 6, with an error in the fit of 0.002 cm^–1^. For excited state energy levels with *J* > 6, a perturbation was present that prevented fitting using the standard asymmetric top Hamiltonian. Based on previous anharmonic frequency calculations and observed vibrational bands of isoprene, the perturbation is most likely caused by Coriolis coupling between the ν_26_ and ν_17_ vibrations or a combination band that lies near the ν_26_ band. The excited state rotational constants from the fit show reasonable agreement with previous anharmonic calculations performed at the MP2/cc-pVTZ level of theory. The jet-cooled spectrum is compared with previous high-resolution measurements of this band at room temperature and shows that understanding the perturbation will be necessary to accurately model this vibrational band.

## 1 Introduction

Isoprene (2-methyl-1,3-butadiene) is one of the simplest conjugated hydrocarbons and is a central molecule in the chemistry of earth’s atmosphere. Isoprene is naturally emitted from plants and is the most abundant nonmethane hydrocarbon in Earth’s atmosphere, with total emissions estimated to be on the order of 500 Tg per year.^1^ The amount of isoprene emitted dominates other nonmethane hydrocarbons and makes accurately measuring and modeling isoprene vital for understanding and predicting atmospheric chemistry.^2^ In the atmosphere, isoprene quickly reacts with hydroxyl radical and ozone; these reactions make isoprene a major factor in the production of ozone^3^ and secondary organic aerosols^4−6^ in the troposphere. In addition, reactions of isoprene affect the oxidative capacity of the troposphere^7,8^ as well as the abundances of many important atmospheric species, including ozone, hydroxyl radical, nitrogen oxide radicals, carbon monoxide, and oxygenated and nitrated organic compounds.^9^ The central importance of isoprene in atmospheric chemistry makes it crucial to continue developing more accurate models of isoprene emission and additional means to monitor its concentration in the atmosphere.

Isoprene is also of interest from a fundamental perspective, as it exhibits interesting conformational properties as one of the simplest conjugated hydrocarbon species. The conformational landscape with respect to rotation about the central C–C single bond is similar to 1,3-butadiene, with the lowest energy conformation being *s*-*trans* with a higher-energy, non-planar *s*-*gauche* conformer lying about 10–15 kJ/mol higher in energy.^10,11^ The higher-energy conformers of 1,3-butadiene^12^ and isoprene^13^ have only recently been experimentally confirmed to be non-planar through observation of their pure rotational spectra.

Spectroscopy of isoprene is an important tool for studying its presence in the atmosphere, its reactions, and its structural properties. Infrared spectroscopy is a particularly promising approach because isoprene has several strong infrared absorption bands (ν_26_, ν_27_, and ν_28_) that lie in the infrared atmospheric window from 8 to 14 μm.^14^ Accurate gas-phase infrared absorption cross-sections of isoprene were measured in 2014 by Brauer et al.,^14^ which have enabled observation of isoprene in the atmosphere using infrared spectroscopy from satellites^2,15,16^ and in human breath using a laser-based spectrometer with a multipass cell.^17^ The satellite measurements currently rely on a pseudo line list for concentration retrievals of isoprene,^16^ making it desirable to obtain a rigorous spectroscopic model that can be applied to measurements at a wide range of temperatures and pressures. Other previous studies of the infrared spectrum of isoprene include an examination of experimental measurements and computational predictions of the infrared and Raman spectra of isoprene,^18^ a high-resolution measurement of the ν_26_ vibrational band,^19^ a measurement of the infrared spectrum of isoprene obtained in noble gas matrices,^20^ and a measurement of the C–H stretching region in helium nanodroplets.^21^ Isoprene has also been studied via microwave spectroscopy,^13,22^ which was used to measure reaction product ratios for the ozonolysis of isoprene in a cryogenic buffer gas cell.^23^

Our research group previously reported the first high-resolution infrared spectrum of isoprene in the region of the ν_26_ vibrational band.^19^ This band is notable because it is one of the strong bands within the infrared atmospheric window, making it potentially useful for measurements of isoprene concentration in the atmosphere or other locations. The room-temperature spectrum was highly congested, but we used the strong Q-branch features along with anharmonic rotational constants predicted at the MP2/cc-pVTZ level of theory to obtain estimated rotational constants for this vibrational band. We tentatively assigned one of the observed peaks as the ν_17_ vibrational band, though this assignment was not consistent with the computed band intensities, as discussed by Ito.^20^ To follow up on our previous work, in this study, we have constructed a supersonic expansion source to measure the high-resolution infrared spectrum of the ν_26_ band of isoprene under jet-cooled conditions. This has alleviated the congestion due to hot bands in the spectrum as well as the congestion from the many populated rotational levels at room temperature. The details of the spectrometer are shared below along with our observation and analysis of the spectrum.

## 2 Experimental Section

High-resolution infrared spectra of jet-cooled isoprene were acquired using a quantum cascade laser (QCL)-based infrared spectrometer coupled to a pulsed supersonic expansion. The components and optical layout of the spectrometer are similar to the instrument described previously by our research group.^17,19^ The major change has been to add a supersonic jet source for introducing samples of interest into the spectrometer instead of using a room temperature multipass cell. Below, we describe the optical layout of the spectrometer, the construction of the supersonic expansion source, and the details of timing the pulsed jet with sweeping the frequency of the cw QCL light source.

**Figure 1:**
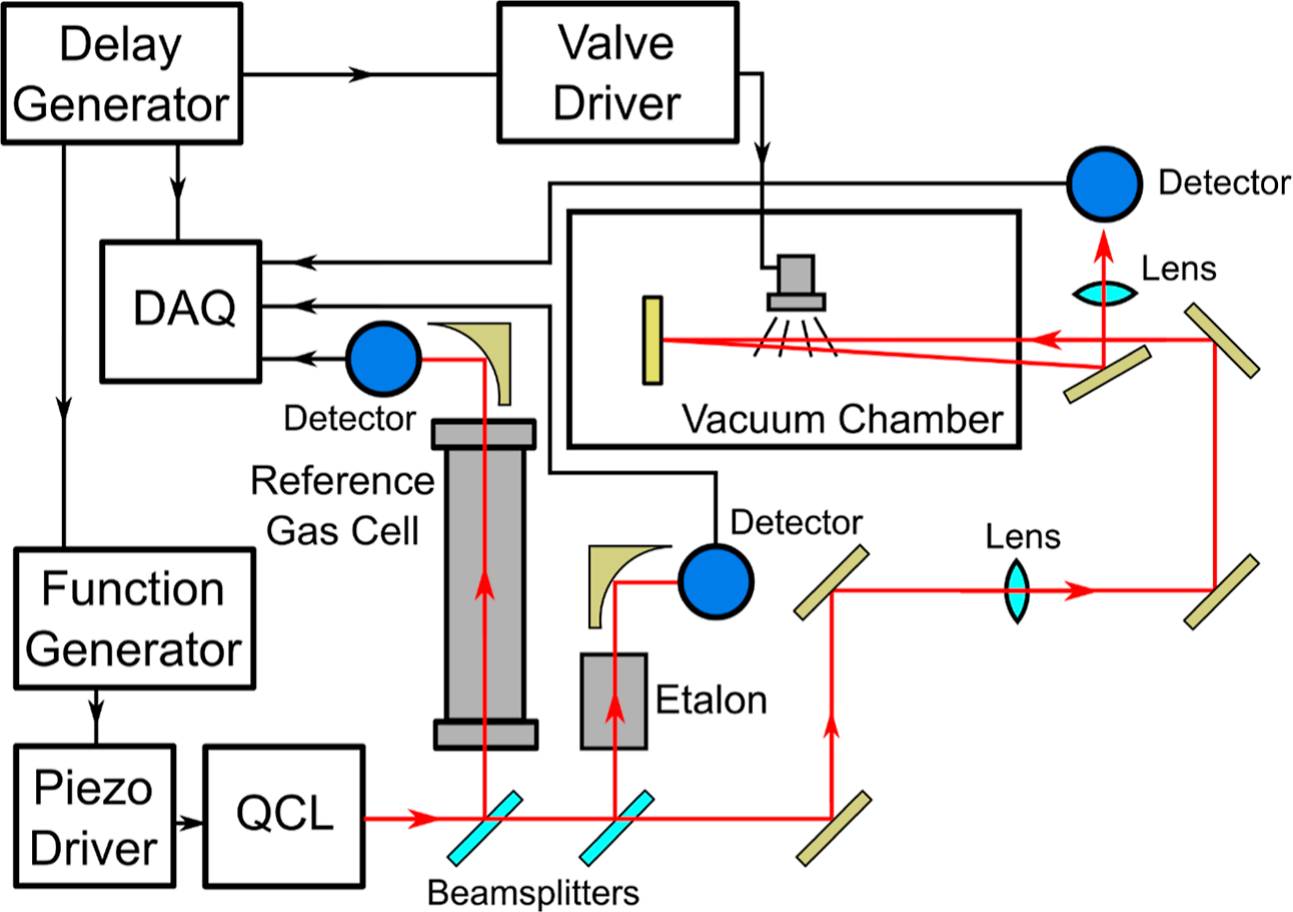
Experimental layout of the spectrometer used in this study. DAQ, data acquisition card; QCL, quantum cascade laser.

The optical layout of the spectrometer is largely unchanged from our previous work^17^ (see Figure 1). In brief, light is provided by an external cavity QCL with a mode-hop free tuning range of 962–1019 cm^–1^ (Daylight Solutions). Light from the laser is sent through two beamsplitters and to a germanium etalon and a reference gas cell containing methanol to provide relative and absolute frequency calibration, respectively. We estimate the accuracy of our frequency calibration to be ∼0.0005 cm^–1^ with this calibration setup by comparing our calibrated methanol spectra to methanol spectra generated using SpectraPlot^24^ (which uses data from the HITRAN database^25^). The remainder of the light is sent through a lens and into the vacuum chamber where the supersonic expansion source is located. The beam is sent through the expansion approximately 7 mm from the exit of the pulse valve, reflected off a mirror inside the vacuum chamber, then sent back through the expansion and out of the vacuum chamber. After exiting the chamber, the light is reflected off of a D-shaped mirror through a focusing lens and onto a thermoelectrically cooled MCT detector. Data from all three detectors (for the etalon, reference gas cell, and supersonic expansion) are digitized using a data acquisition card (DAQ, Measurement Computing, USB-1808X) and then saved onto a computer using a custom Python program. After saving the data, the frequency axis of each spectrum was calibrated using the data from the etalon and methanol reference cell.

The major change in this work compared to our previous studies of isoprene is the introduction of a supersonic expansion source to allow us to measure low-temperature spectra of isoprene. Isoprene was introduced into the spectrometer by bubbling helium gas through a sample of liquid isoprene (Acros Organics, 98% purity). The He/isoprene mixture was then sent through a pulse valve with a 0.5 mm diameter pinhole (Parker-Hannefin, Miniature High Speed Vacuum Dispense) and expanded into a vacuum chamber. The chamber was evacuated by a diffusion pump (National Research Corporation, 0184) backed by a mechanical pump (Welch Duo-Seal model 1397). The pressure in the chamber was approximately 10^–5^–10^–4^ torr, monitored by a cold cathode gauge (Varian, 524-2). The pressure of the He/isoprene mixture leading into the valve was maintained at about 1000 torr for the experiments described in this work. The pulse valve was run at a repetition rate of 5 Hz with a pulse duration of 2.0 ms.

**Figure 2:**
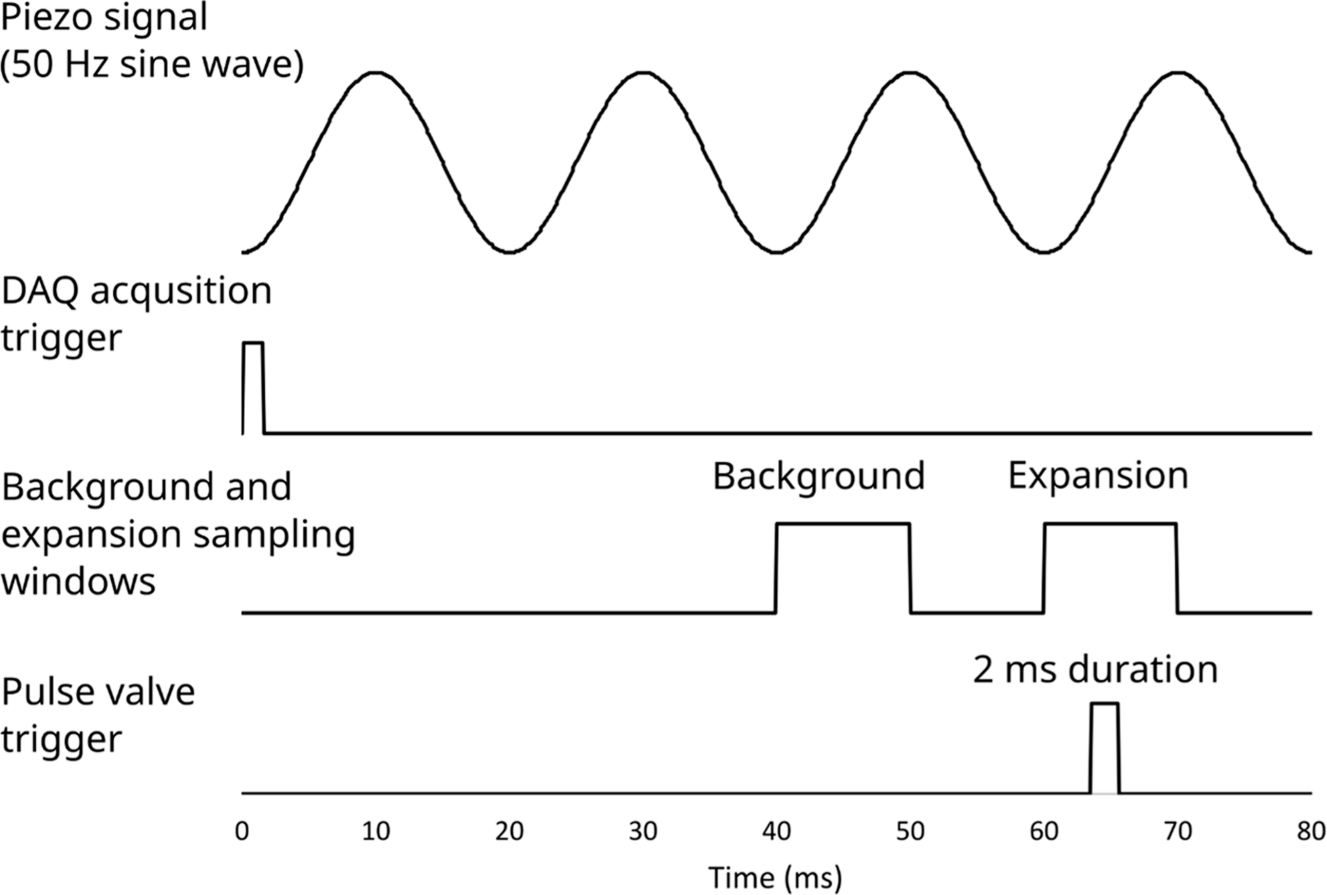
Depiction of the timing used for the experiment. The first trigger is sent (at time 0) to a function generator to begin producing a 50 Hz sine wave signal, which is amplified by a piezo driver and sent to the laser to sweep the laser frequency. After a slight delay (0.5 ms), a trigger signal is sent to the data acquisition card (DAQ) to begin recording data from the detectors. The background signal is acquired in a sampling window from 40 to 50 ms after the trigger is sent to begin sweeping the laser frequency, before the pulse valve opens. The expansion signal is acquired in a sample window from 60 to 70 ms from the start of the timing sequence. The third trigger is sent to the pulse valve driver at time = 63.57 ms and the valve is opened for 2 ms.

The timing of the experiment is shown in Figure 2 and was primarily controlled using a delay generator (Stanford Research Systems, DG535). The timing sequence for our experiment was modified from the segmented rapid-scan scheme used by Luo et al.^26^ The delay generator sends triggers to three different pieces of equipment: a function generator (Siglent, SDG1025), which feeds into a piezo driver (ThorLabs, MDT694B) and controls the frequency sweeping of the laser; the DAQ; and the pulse valve driver (Parker Hannefin, Iota One), which controls the opening and closing of the pulse valve used to generate the supersonic expansion. The first trigger is sent to the function generator; this begins a burst of four sinusoidal sweeps of the PZT in the QCL, which sweeps the laser frequency by about 0.7–0.8 cm^–1^ per sweep. The second signal is sent to the DAQ to trigger acquisition of the data from the detectors by the Python program used to control the experiment. The third signal is sent after a delay of 63.57 ms to the pulse valve driver. The opening of the pulse valve and formation of the supersonic expansion is timed so that it occurs during the fourth and final sweep of the laser frequency. The data acquisition program is set to record two windows of data; the first is a background signal recorded during the third sweep of the laser frequency and the second is the sample signal recorded during the fourth sweep of the laser frequency while the pulse valve is open and the supersonic expansion is present in the vacuum chamber. (The data from the etalon and reference gas cell are also collected in this sampling window). The data from the sample and background windows are used to calculate the absorbance in the supersonic jet. This process is repeated and the results are averaged and calibrated. Spectra presented in this paper were generated from 500 averages for each section of the spectrum. After calibration, we extract only the portion of the spectrum that coincides with the opening of the pulse valve. To decrease background interference, spectra were also acquired in the same manner without the pulse valve being turned on and the absorbance calculated for these spectra were subtracted from the spectra obtained with the pulse valve on. Using this timing scheme, we obtain about 0.15 cm^–1^ of the jet-cooled spectrum of isoprene at a time. The laser frequency was then changed by about 0.1 cm^–1^, and the process was repeated to obtain the next portion of the spectrum, with some overlap between each section of the spectrum. This process was repeated and the individual sections of the spectrum were combined into a single jet-cooled spectrum of isoprene from 985.5 to 999 cm^–1^. (The combined spectrum is available in the Supporting Information).

## 3 Results

**Figure 3:**
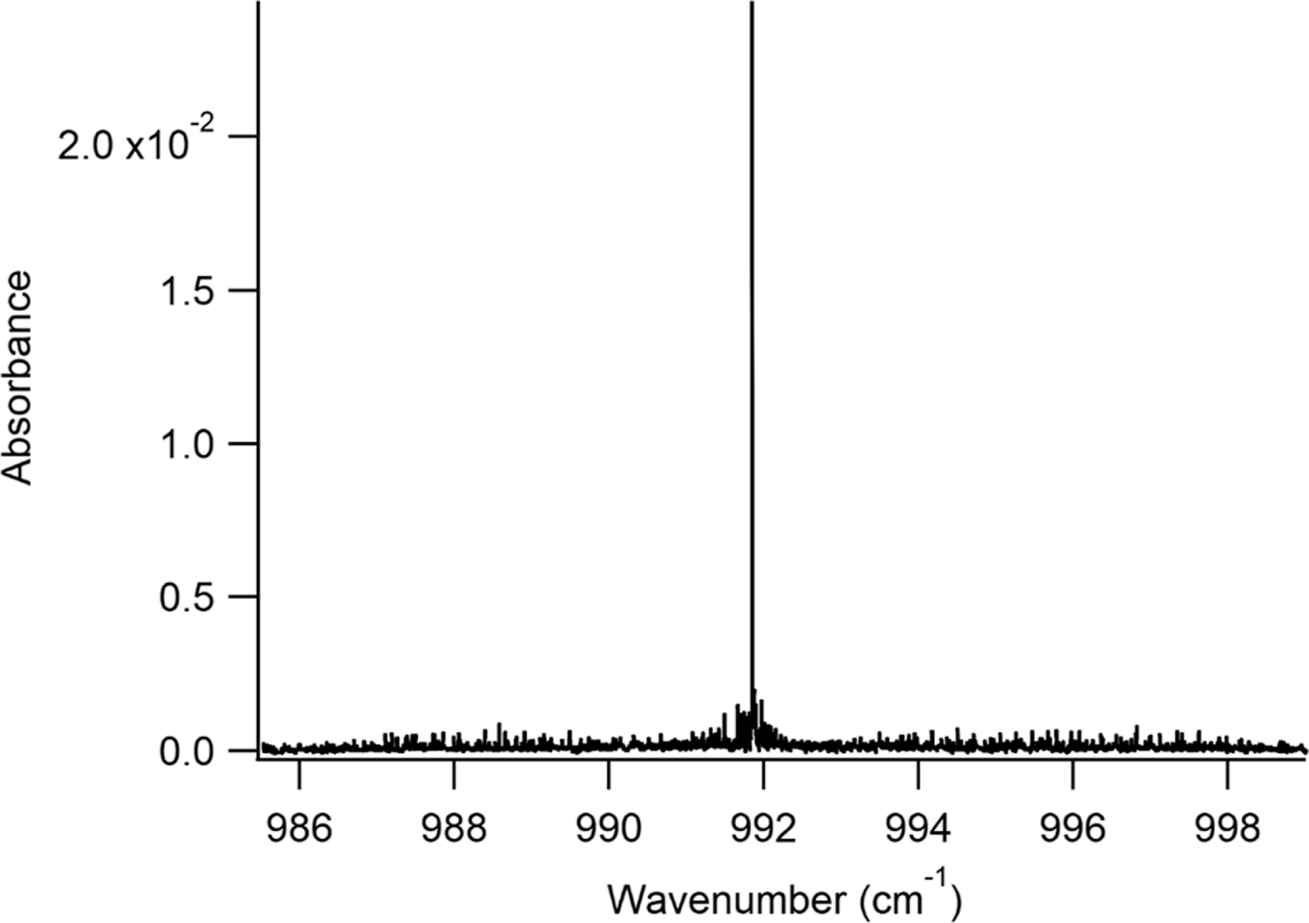
Overview of the jet-cooled spectrum of isoprene from 985.5 to 999 cm^–1^.

**Figure 4:**
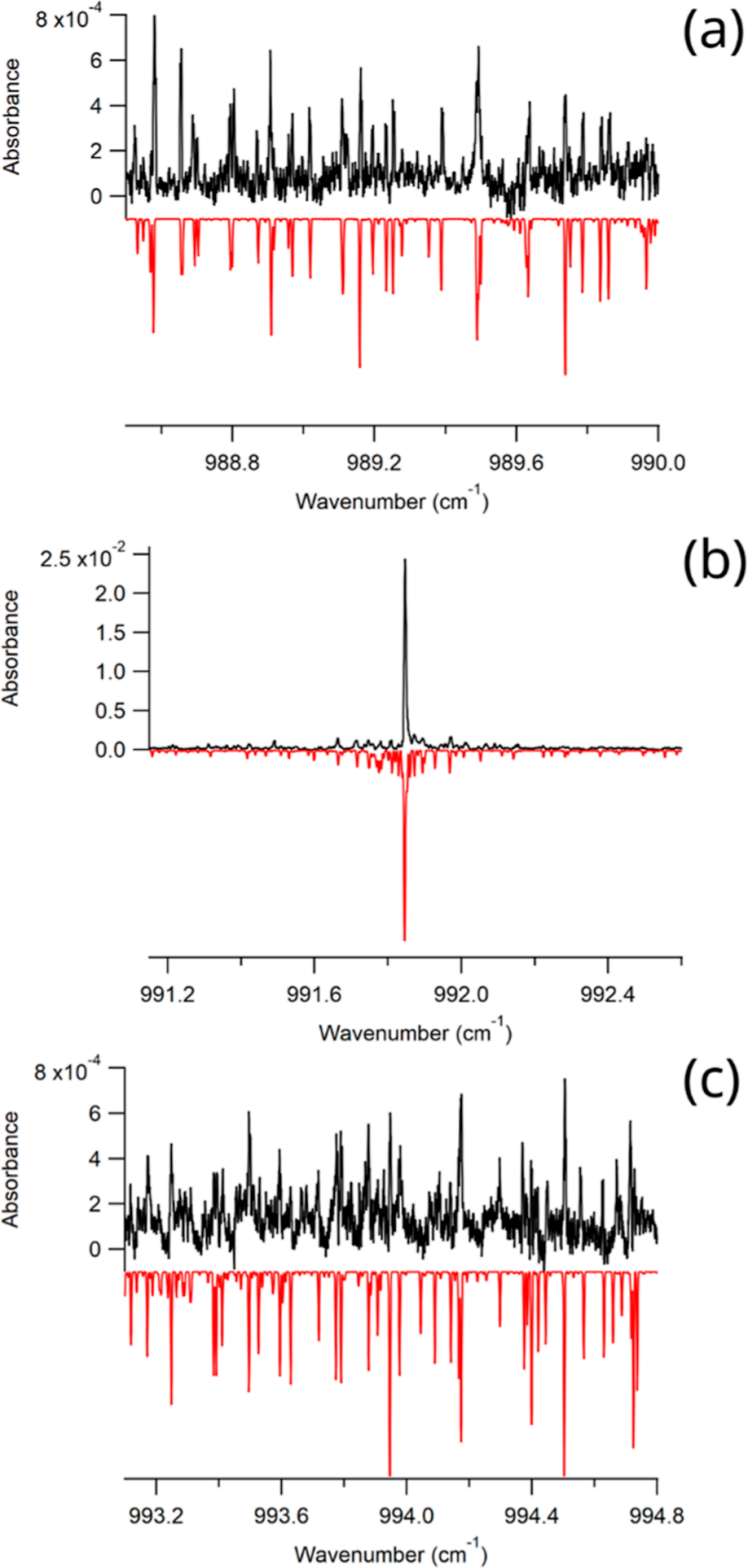
Zoomed-in views of the experimental isoprene spectrum (top, black) and simulated spectrum generated using PGOPHER^27^ (bottom, red). Panel (a) is a portion of the P-branch, panel (b) is the Q-branch region, and panel (c) is a portion of the R-branch. The simulation uses a rotational temperature of 10 K and a linewidth of 0.0035 cm^–1^.

Figure 3 presents an overview of the jet-cooled spectrum of isoprene. As can be seen in the figure, the spectrum is dominated by a strong Q-branch feature at 991.85 cm^–1^, which lies at the same frequency as the second-strongest Q-branch feature observed in the high-resolution room temperature spectrum and is due to the ν_26_ vibrational mode.^19^ On either side of the Q-branch feature are well-resolved ro-vibrational features with significantly reduced congestion compared to the room temperature spectrum. A closer view of three regions of the spectrum in the P-, Q-, and R-branches can be seen in Figure 4, which illustrates our ability to observe individual, well-resolved peaks with our spectrometer.

**Figure 5:**
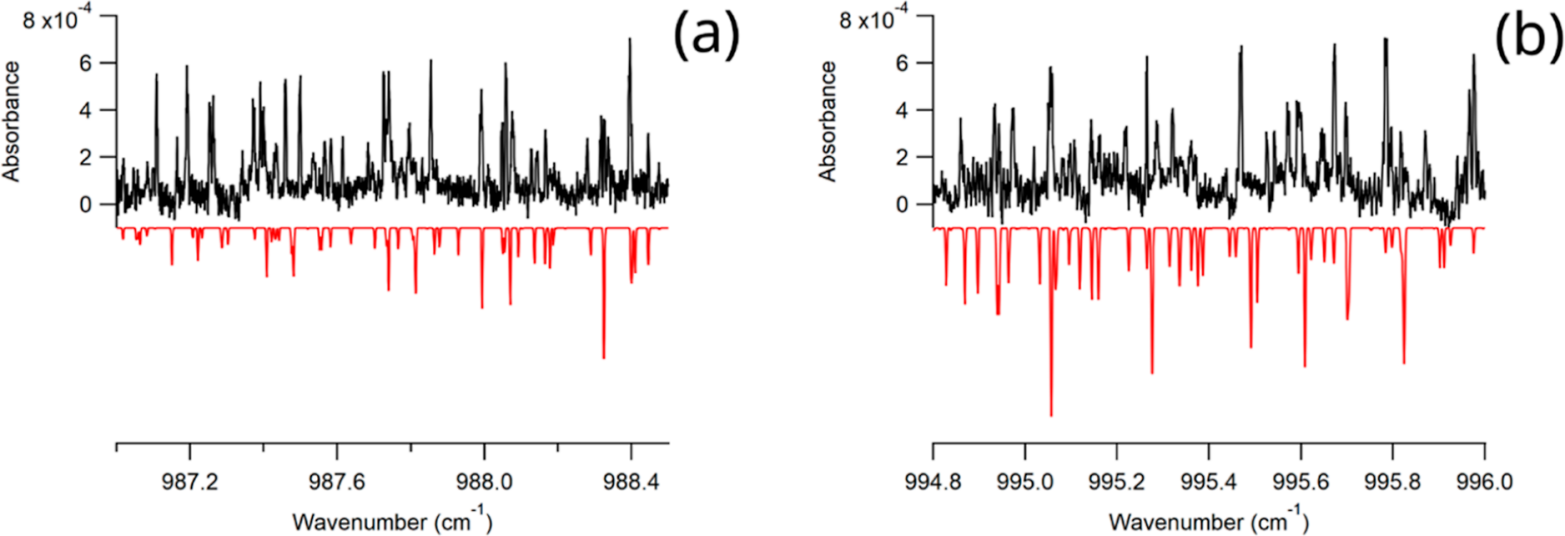
Zoomed in views of the experimental isoprene spectrum (top, black) and simulated spectrum generated using PGOPHER^27^ (bottom, red). Panel (a) is a portion of the P-branch and panel (b) is a portion of the R-branch. These regions include transitions to upper state energy levels with *J* > 6 and show the discrepancy between the experimental spectrum and the simulation, as well as additional peaks that do not appear in the simulated spectrum. The simulation uses a rotational temperature of 10 K and a linewidth of 0.0035 cm^–1^.

The spectrum was fit to an asymmetric top Hamiltonian (using the S reduction and *I*^*r*^ representation) with PGOPHER.^27^ The ground state rotational constants were fixed to values obtained from microwave spectroscopy^23^ while the upper state constants were obtained from assigning and fitting our spectrum. We fit only the *A*′, *B*′, and *C*′ constants; the values for the distortion constants were fixed to the ground state values as including them in the fit did not have a substantial effect on the error of the fit. In the process of fitting our spectrum, we found good agreement between our simulation and the experimental spectrum up to a value of *J* = 6 in the ν_26_ state, as can be seen in the simulations included in Figure 4. For transitions to upper state levels with *J* ≥ 7, we saw a noticeable deviation of the experimental peaks from the simulation and additional peaks that were not predicted by the simulation, as can be seen in Figure 5. Including these transitions in our fit greatly increased the error in the fit and could not be compensated by including distortion constants in the fit. The molecular constants obtained from the fit to transitions with upper state *J* values ≤6 are included in Table 1 and the full linelist of assigned transitions is included in Table S1 of the Supporting Information. The error in the fit is 0.0021 cm^–1^, which is acceptable, but somewhat higher than our estimated frequency accuracy of 0.0005 cm^–1^, indicating that even for this limited set of transitions, there is likely a perturbation causing a small deviation from a fit to the standard asymmetric top Hamiltonian.

**Table 1:** Molecular Constants (in cm^–1^) Obtained for the ν_26_ Band of Isoprene from This Work, Along with the Ground State Constants from Microwave Spectroscopy,^23^ Rotational Constants Calculated at the MP2/cc-pVTZ Level of Theory,^19^ and Approximate Constants Obtained from a Previous Contour Fit of the Room Temperature Q-Branch^19^

	ground state^23^	ν_26_ (this work)^a^	ν_26_ (calculated)^19^	ν_26_ (contour fit)^19^
ν_0_		991.8621(5)	1004.9	991.875
*A*	0.28443161(3)	0.28318(3)	0.284075	0.275224
*B*	0.13927088(3)	0.13697(3)	0.139018	0.141163
*C*	0.09513782(3)	0.09525(4)	0.095119	0.095169
*D*_J_ (×10^–7^)	0.12(1)	0.12^b^		
*D*_JK_ (×10^–7^)	1.80(2)	1.80^b^		
# of transitions		103		
error		0.0021		

a The fit only included transitions to upper state levels with *J* ≤ 6.

b Upper state distortion constants were fixed to their ground state values.

## 4 Discussion

We can gain additional insights about the ν_26_ band of isoprene by comparing the jet-cooled spectrum from this work to the previously measured high-resolution room temperature spectrum of this region.^19^ The first major difference is the lack of any other strong features in the jet-cooled spectrum besides the strong ν_26_ band. In the room temperature spectrum, there were several additional strong features in the Q-branch region that were mostly attributed to hot bands, which are not expected to appear in the jet-cooled spectrum. However, one feature at 991.37 cm^–1^ was tentatively assigned as the ν_17_ band of isoprene. This assignment was made because a similar peak appeared in a matrix-isolation spectrum of isoprene,^20^ which may have indicated that the peak was due to a transition from the vibrational ground state instead of a hot band. This assignment was tentative because according to quantum chemistry calculations, the intensity of the ν_17_ should be much lower than what was observed in both the matrix and gas-phase spectra. As can be seen in Figure 4b, we see no evidence of a strong peak anywhere near 991.37 cm^–1^, which confirms that this peak in the room temperature spectrum is not due to the ν_17_ vibrational mode, in agreement with the calculations of the band intensity. The peak in the room temperature spectrum is almost certainly due to a hot band, which is a better match for its relative intensity compared to the main ν_26_ feature.

**Figure 6:**
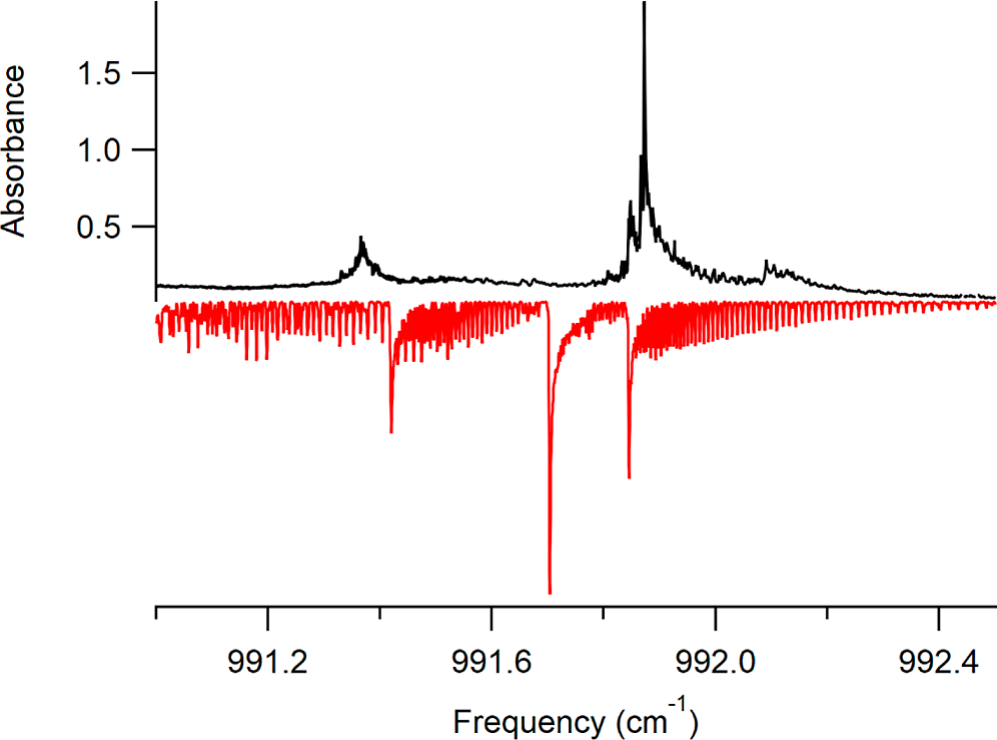
Comparison of the previously recorded high-resolution room temperature spectrum of isoprene^19^ (top, black) to a simulation using the molecular constants reported in Table 1 at a temperature of 300 K (bottom, red).

The second item of note when comparing the jet-cooled spectrum to the room temperature spectrum is that the molecular constants we have obtained from the jet-cooled spectrum do not provide an accurate simulation of the room temperature spectrum. In Figure 6, we compare the high-resolution room temperature spectrum to a simulated spectrum generated using PGOPHER at a temperature of 300 K using the constants from Table 1. As seen in the figure, the only part of the spectrum that agrees between the experiment and the simulation is the feature at 991.85 cm^–1^. It is especially striking that the strongest peak in the experimental spectrum at 991.87 cm^–1^ is not reproduced in the simulation, though there is a peak of similar relative intensity at 991.70 cm^–1^ in the simulated spectrum. The disagreement between the experimental spectrum and the simulation is driven by the perturbation that we observed when fitting the jet-cooled spectrum.

As noted above, we were only able to obtain a satisfactory fit for the jet-cooled spectrum by including transitions to energy levels with *J*′ = 6 or lower. Peaks for higher *J*′ values were present in the spectrum but were shifted from their expected positions. We also observed additional peaks that were not reproduced in the simulated spectrum, which leads us to conclude that there is an additional vibrational state interacting with and perturbing the ν_26_ state. We have not yet been able to assign the extra peaks and perform a deperturbation analysis, but this will be necessary to accurately model the ν_26_ band at room temperature (or any other temperature that might be desired). Having an accurate model of the spectrum would enable simulation of isoprene absorption at a variety of temperatures and pressures and enable using infrared spectra of isoprene for concentration retrievals in a variety of physical conditions. At present, the concentration retrievals from infrared satellite measurements^16^ rely on a pseudo line list for isoprene instead of a rigorous spectroscopic model.

At this point, we can only speculate on the identity of the perturbing state. The only fundamental band that lies close to ν_26_ is the ν_17_ band. Previous anharmonic frequency calculations of these bands^18−20^ predict that ν_17_ should lie within about 10 cm^–1^ or so of ν_26_, but that the two vibrations belong to different symmetries within the *C*_*s*_ point group (ν_26_ is A″, ν_17_ is A′). Because these two states are predicted to lie so close in energy, it is possible that Coriolis coupling between ν_26_ and ν_17_ could explain the perturbation. Our previous anharmonic frequency calculations (at the MP2/cc-pVTZ level of theory, see ref (19)) give predicted Coriolis coupling constants along the *a*, *b*, and *c* axes of ζ_17,26_^*a*^ = −0.05051, ζ_17,26_^*b*^ = −0.24022, and ζ_17,26_^*c*^ = 0 cm^–1^ and these predicted values may provide a starting point for future analyses. Another possibility for the identity of the perturbing state is a combination band. In our previous anharmonic calculations, the only combination that was close to the ν_26_ band was the ν_19_ + ν_32_ combination band, which was predicted at 999 cm^–1^. This combination band would have the correct symmetry to interact with ν_26_ (ν_19_ has A′ symmetry and ν_32_ has A″ symmetry, meaning the combination will have A″ symmetry). If we use the experimental frequencies for ν_19_ and ν_32_ instead of the calculated frequencies, the band is expected to lie significantly lower in frequency (∼976 cm^–1^), making this assignment less likely. Two other possible combination bands that would lie near ν_26_ based on experimental vibrational frequencies are 2ν_31_ + ν_32_ (∼1001 cm^–1^) and ν_31_ + 3ν_32_ (∼999 cm^–1^), though, at present, we have no evidence to favor one over the other.

Finally, we can compare the rotational constants obtained from our limited fit of ν_26_ to the calculated anharmonic rotational constants at the MP2/cc-pVTZ level and the approximate rotational constants we obtained from fitting the Q-branch contour in our previous work.^19^ The calculated and approximate constants are included in Table 1 for comparison with the constants obtained from the fit. The agreement between the experimental and calculated *A* and *C* constants is better than 0.5% (0.3% difference for *A* and 0.1% difference for *C*) but the agreement for the *B* constant is significantly worse (1.5% difference). When comparing to the contour fit, the agreement is much worse for *A* and *B* (∼3% difference for these constants) though the *C* constant matches quite well between the jet-cooled data and the contour fit (<0.1% difference). The mismatch between the contour fit and the fit of the jet-cooled data is unsurprising, given the obvious perturbations we have observed in the spectrum at higher *J* values. An understanding of the perturbation will be necessary to accurately interpret the spectral features of the room temperature spectrum of isoprene in this spectral region, which will aid in modeling the spectrum for use in sensing applications.

## 5 Conclusions

In this article, we have presented the first supersonic jet-cooled infrared spectrum of isoprene, a key molecule in atmospheric chemistry. We have observed and assigned the ν_26_ band near 992 cm^–1^, which is one the strongest absorption bands of isoprene and lies in the infrared atmospheric window. We obtained excited state rotational constants for this vibrational level, though we could only fit transitions up to *J* = 6 in the excited state due to a perturbation affecting this band. The perturbation is likely caused by Coriolis coupling between ν_26_ and ν_17_ or interaction with a combination band, though we have not yet conclusively identified the exact cause. The experimental rotational constants agree well with previously calculated constants for this vibrational energy level calculated at the MP2/cc-pVTZ level of theory, though the *B* rotational constant has a larger deviation than the other two rotational constants. Simulations of the vibrational band at room temperature show poor agreement with previously measured high-resolution room temperature spectra, showing the need to understand and assign the perturbation to accurately model this vibrational band for future use in isoprene concentration measurements.

## Supporting Information Available

List of assigned transitions in the fit with observed and calculated frequencies (PDF)
PGOPHER file used to analyze the spectrum (ZIP)
Text file containing the combined, calibrated spectrum of jet-cooled isoprene (TXT)
