# Supplementary material for: Rotationally Resolved Infrared Spectroscopy of Supersonic Jet-Cooled Isoprene
Source: J Phys Chem A. 2023 May 26;127(22):4873–9. doi: 10.1021/acs.jpca.3c02272 (PMC10258839; doi:10.1021/acs.jpca.3c02272)
Supplement: Supplementary file 1 — jp3c02272_si_001.pdf [file jp3c02272_si_001.pdf]

Rotationally-Resolved Infrared Spectroscopy of Supersonic Jet-Cooled Isoprene

Jacob T. Stewart*, Lauren Hino, Carter Pavlonnis, Katarina R. Reyna, Binh L. N. Vo

Department of Chemistry, Connecticut College, New London, CT 06320

Table S1: List of assigned transitions, wavenumbers of the peaks observed in the experimental spectrum, calculated wavenumbers based on the simulated spectrum, and the difference between the observed and calculated values. All values are given in cm^{-1}. The transitions are labeled with the following notation: ΔK_a ΔJ K_a'',K_c''(J''). For example, pP7,0(7) is the transition from J''= 7 K_a''=7 K_c''=0 in the ground vibrational state to J'=6 K_a'=6 K_c'=0 in the excited vibrational state.

Transition	Observed	Calculated	Obs - Calc
pP7,0(7)	987.9914	987.9936	-0.0022
pP7,1(7)	987.9914	987.9936	-0.0022
pP6,1(7)	988.3256	988.3249	0.0007
pP6,2(7)	988.3256	988.3249	0.0008
pP6,0(6)	988.5800	988.5773	0.0027
pP6,1(6)	988.5800	988.5773	0.0027
pP5,2(7)	988.6541	988.6586	-0.0045
pP5,3(7)	988.6541	988.6553	-0.0012
pP5,1(6)	988.9063	988.9092	-0.0029
pP5,2(6)	988.9063	988.9085	-0.0022
pP4,4(7)	988.9679	988.9689	-0.0010
pP4,3(7)	989.0191	989.0189	0.0002
pP5,0(5)	989.1611	989.1587	0.0025
pP5,1(5)	989.1611	989.1586	0.0025
pP3,5(7)	989.1942	989.1952	-0.0010
pP4,3(6)	989.2349	989.2329	0.0020
pP4,2(6)	989.2537	989.2521	0.0016
pP2,6(7)	989.2780	989.2772	0.0007
pP1,6(7)	989.3542	989.3529	0.0013
pP3,4(7)	989.3912	989.3879	0.0033
pP4,2(5)	989.4916	989.4879	0.0037
pP2,5(7)	989.4916	989.4900	0.0016
pP2,5(6)	989.6303	989.6285	0.0018
pP3,3(6)	989.6351	989.6329	0.0023
pP4,1(4)	989.7370	989.7371	0.0000

pP4,0(4)	989.7396	989.7379	0.0017
pP1,5(6)	989.7504	989.7518	-0.0014
pP3,3(5)	989.7873	989.7858	0.0015
pP2,4(6)	989.8379	989.8361	0.0018
pP3,2(5)	989.8605	989.8598	0.0006
pP2,4(5)	989.9671	989.9661	0.0010
pP3,2(4)	990.0554	990.0552	0.0002
pP3,1(4)	990.0876	990.0848	0.0028
pP2,3(5)	990.1470	990.1463	0.0007
pP1,4(5)	990.1470	990.1437	0.0033
pP2,3(4)	990.2889	990.2867	0.0023
pP3,1(3)	990.3136	990.3106	0.0030
pP3,0(3)	990.3171	990.3173	-0.0002
pP2,2(4)	990.4249	990.4234	0.0015
pP1,3(4)	990.5143	990.5132	0.0011
pP2,2(3)	990.5893	990.5879	0.0014
pQ4,3(6)	990.7136	990.7151	-0.0016
pQ3,3(6)	990.7822	990.7814	0.0008
rP1,5(6)	990.8165	990.8194	-0.0029
pP1,2(3)	990.8485	990.8510	-0.0025
pP2,1(2)	990.8675	990.8684	-0.0009
pP2,0(2)	990.8979	990.9015	-0.0036
pQ3,2(5)	990.8979	990.9017	-0.0038
pQ3,0(3)	991.0058	991.0062	-0.0004
rP1,4(5)	991.0058	991.0103	-0.0045
pQ2,2(4)	991.0058	991.0063	-0.0005
pQ3,2(4)	991.0799	991.0801	-0.0003
pQ2,0(2)	991.2813	991.2825	-0.0012
rP0,4(4)	991.3110	991.3110	-0.0001
pQ2,5(6)	991.6629	991.6642	-0.0013
pQ1,1(1)	991.7126	991.7147	-0.0022
pQ1,2(2)	991.7464	991.7468	-0.0004
pQ1,3(3)	991.7771	991.7821	-0.0050
pQ1,4(4)	991.8079	991.8109	-0.0030
rQ0,5(5)	991.8724	991.8719	0.0006
rQ0,4(4)	991.8941	991.8940	0.0002
rQ0,3(3)	991.9287	991.9276	0.0012
rQ0,2(2)	991.9710	991.9686	0.0023
rQ1,5(6)	991.9710	991.9679	0.0031
rQ1,3(4)	992.1413	992.1414	-0.0001

rQ1,2(3)	992.2223	992.2237	-0.0014
rR0,0(0)	992.2769	992.2822	-0.0053
pR1,1(2)	992.2769	992.2817	-0.0048
rQ1,1(2)	992.2900	992.2904	-0.0004
pR3,3(5)	992.2900	992.2892	0.0009
rQ2,4(6)	992.3764	992.3784	-0.0020
rQ2,3(5)	992.4941	992.4951	-0.0010
rR1,0(1)	992.8127	992.8111	0.0016
rQ3,2(5)	992.9487	992.9528	-0.0041
rR1,1(2)	993.0242	993.0241	0.0001
rR1,2(2)	993.1183	993.1195	-0.0012
rR0,3(3)	993.1740	993.1709	0.0032
rR1,2(3)	993.2482	993.2486	-0.0004
rR2,0(2)	993.3849	993.3837	0.0012
rR2,1(2)	993.3929	993.3914	0.0016
rR1,3(3)	993.4124	993.4102	0.0021
rR1,3(4)	993.4981	993.4962	0.0019
rR0,4(4)	993.5290	993.5268	0.0022
rR2,1(3)	993.5937	993.5952	-0.0016
rR2,2(3)	993.6292	993.6301	-0.0010
rR1,4(4)	993.7165	993.7193	-0.0028
rR1,4(5)	993.7763	993.7745	0.0018
rR2,2(4)	993.7908	993.7901	0.0007
rR2,3(4)	993.8783	993.8788	-0.0005
rR3,0(3)	993.9477	993.9461	0.0016
rR3,1(3)	993.9477	993.9471	0.0006
rR2,3(5)	993.9776	993.9774	0.0002
rR1,5(5)	994.0439	994.0453	-0.0014
rR2,4(5)	994.1395	994.1412	-0.0017
rR3,2(4)	994.1730	994.1731	-0.0001
rR3,2(5)	994.3710	994.3753	-0.0043
rR3,3(5)	994.3983	994.3996	-0.0013
rR4,1(4)	994.5059	994.5030	0.0028
rR4,0(4)	994.5059	994.5029	0.0029
rR4,2(5)	994.7262	994.7248	0.0014
rR4,1(5)	994.7262	994.7239	0.0023
rR5,1(5)	995.0555	995.0568	-0.0014
rR5,0(5)	995.0555	995.0568	-0.0014
